# Identifying central symptom clusters and correlates in children with acute leukemia undergoing chemotherapy: a network analysis

**DOI:** 10.3389/fonc.2023.1236129

**Published:** 2023-08-21

**Authors:** Jia Fang, Cho-Lee Wong, Chun-Qin Liu, Hai-Ying Huang, Yi-Shu Qi, Li-Ling Xu, Mei-Xiang Wang, Yan Lin

**Affiliations:** ^1^ Department of Nursing, Guangzhou Women and Children’s Medical Center, Guangzhou Medical University, Guangdong Provincial Clinical Research Center for Child Health, Guangzhou, China; ^2^ Nethersole School of Nursing, Faculty of Medicine, The Chinese University of Hong Kong, Hong Kong, Hong Kong SAR, China; ^3^ School of Nursing, Guangzhou Medical University, Guangzhou, China; ^4^ School of Nursing, Guangdong Pharmaceutical University, Guangzhou, China

**Keywords:** acute leukemia, children, network analysis, symptom cluster, symptoms management

## Abstract

**Background:**

Previous studies have examined symptom clusters in children with acute leukemia, yet a knowledge gap persists regarding central symptom clusters and their influencing factors. By identifying these central clusters and associated factors, healthcare providers can enhance their understanding and effective management of symptoms. Our study seeks to address this gap by identifying symptom clusters, exploring central clusters, and investigating the demographic and health-related factors associated with these clusters in children with acute leukemia undergoing chemotherapy.

**Methods:**

A total of 586 children with acute leukemia from January 2021 to April 2023 were recruited from China. They were investigated using Memorial Symptom Assessment Scale 10-18 during chemotherapy. The principal component analysis was used to identify the symptom clusters. An association network was conducted to describe the relationships among symptoms and clusters. A multiple linear model was used to investigate the associated factors for the severity of overall symptoms and each symptom cluster.

**Results:**

Five clusters were identified, including oral and skin cluster, somatic cluster, self-image disorder cluster, gastrointestinal cluster and psychological cluster. Gastrointestinal cluster was the most central symptom cluster. Age, sex, clinical classification, number of having chemotherapy and education degree and marital status of the primary caregiver are associated with the severity of these five symptom clusters.

**Conclusion:**

Our study highlights the importance of evaluating symptom clusters in children with acute leukemia during chemotherapy. Specifically, addressing gastrointestinal symptoms is crucial for effective symptom management and overall care.

## 1 Introduction

Annually, around 400,000 children and adolescents aged 0-19 are diagnosed with cancer, with acute leukemia (AL) being the most common type, accounting for nearly 1 in 3 cases (1–3). In 2021, the World Health Organization (WHO) initiated the global campaign “Increasing Accessibility, Enhancing Quality, Saving Lives” to improve the survival rate and quality of life (QoL) for children with cancer (3). However, China faces significant challenges in this regard, with a staggering 2 million leukemia patients and an annual addition of 75,000 new cases, most of which are children (4, 5). Encouragingly, advancements in pediatric oncology have improved chemotherapy, supportive care, and personalized medicine, resulting in a survival rate of over 90% for children with acute lymphoblastic leukemia. However, combined chemotherapy still causes multiple adverse symptoms (6, 7). Previous studies have found that children with cancer often experience 10–16 symptoms simultaneously (8–11). Furthermore, interactions between symptoms can produce synergistic effects, worsening each other and leading to a decline in QoL and survival time (10, 12). Fatigue/pain/insomnia/depression and nausea/vomiting/lack of appetite are the most common cancer-related symptoms with synergistic effects in cancer survivors (13, 14). Comprehending the interactions among multiple symptoms is crucial to assist healthcare providers in managing symptoms and preventing the emergence of related symptoms.

Identifying symptom clusters is a classic approach for reducing dimensions in real-world clinical practice aimed at simplifying complex symptom interactions (14). According to Kim et al., a “ symptom cluster ” denotes the simultaneous occurrence of two or more symptoms, which may or may not share the same etiology (15). After an exhaustive search, we found only seven studies identifying symptom clusters in children with cancer (9, 11, 16–20). In studies conducted by Yeh et al. (9) and Hockenberry et al. (19, 20), five symptom clusters were identified among 144 and 67 children with cancer, including dry mouth/itching/diarrhea/tingling in hands or feet/change in skin/feeling nervous/I don’t look like myself, dyspnea/dizziness/swelling of arms or legs/cough/problems with urination, lack of concentration/insomnia/lack of energy/feeling drowsy/feeling sad/worrying/feeling irritable/sweating, weight loss/hair loss/mouth sores/constipation/difficulty swallowing, pain/nausea/vomiting/lack of appetite. However, as these three studies collected data from different cancer types (leukemia, lymphoma, or solid tumors), they may not fully capture the symptom burden specifically experienced by children with AL.

Four studies have evaluated symptom clusters in children with AL (11, 16–18). Besides the symptom clusters identified in previous studies in children with cancer, several new symptom clusters have been found among AL, which includes neurological symptom clusters (dizziness and headache) (11, 16). The emergence of a novel symptom cluster may be related to the maintenance of chemotherapy in preventing central nervous system leukemia, which may induce central nervous system toxicity reactions through the disruption of folate homeostasis and/or direct damage to neurons (11). However, previous studies (11, 16–18) on this topic have shown heterogeneity in the number and composition of symptom clusters. The variability in symptom combinations within clusters can be attributed to factors such as the selection of symptoms, statistical methods, and covariates (21, 22). Furthermore, a notable gap exists in the literature regarding the relationship between symptom clusters, hindering the development of targeted interventions. While symptom clusters facilitate the categorization of symptoms, the specific cluster(s) that should receive primary intervention focus remains uncertain. This uncertainty hinders healthcare providers from understanding and developing effective intervention strategies.

The lack of conclusive findings regarding central symptom clusters and their relationships during chemotherapy in children with AL motivated us to explore the central symptom clusters and their interrelationships in this population. Network analysis is a novel approach to visualizing symptoms and symptom clusters, which will not only help researchers identify central symptoms and clusters but also explore symptom mechanical indicators such as centrality (23, 24). To the best of our knowledge, four studies have conducted network analyses to capture complex relationships among symptoms of various cancer survivors (14, 25–27). Three cross-section studies including 3691 cancer survivors revealed that fatigue (26, 28) and lack of appetite (25) were the most central symptoms in the symptom network. Another study including 1078 cancer survivors conducted by Zhu et al. indicated that distress and sadness were the core symptoms in cancer survivors (14). Identifying the central symptom provides healthcare providers with a broad perspective to develop precise intervention strategies. However, the specific symptom cluster that is most central to cancer patients remains unclear, impeding healthcare providers and researchers from fully understanding and developing targeted interventions. By identifying and understanding central symptom clusters using network analysis, healthcare providers can enhance the efficiency and precision of symptom management interventions.

Prior studies have indicated an association between clinical factors, particularly chemotherapy stages, and symptom clusters (11, 29). However, the connection between symptom clusters and demographic factors has not been definitively established in current research (11, 29). Consequently, there is a lack of understanding regarding the factors influencing symptom clusters. To address the existing knowledge gap, our study seeks to answer three key research questions in children with AL undergoing chemotherapy: (1) How many symptom clusters exist? (2) Which symptom cluster is most central? (3) What demographic and health-related factors are associated with these symptom clusters?

## 2 Materials and methods

### 2.1 Study design and setting

We conducted a cross-sectional study through a convenience sample between January 2021 and April 2023. We collected data from 586 children diagnosed with acute leukemia at Guangzhou Women and Children’s Medical Center, which is a National Pediatric Regional Medical Center (Central-South) in China. The medical center has a capacity of 2,400 beds and treats over 200 new cases of leukemia in children annually.

### 2.2 Study population

Eligibility criteria included: 1) participants aged between 3-18 years; 2) diagnosed with AL; 3) within one of the chemotherapy stages, including induction, intensified consolidation, or maintenance phase; 4) willing to provide written informed consent. Participants were excluded if they were: 1) diagnosed with mental illness or cognitive impairment; or 2) had serious complications such as heart, lung, or functional brain failure or undergoing bone marrow transplantation. Primary caregivers who can read, write and comprehend Chinese and who have expressed willingness to provide informed consent were also eligible for participation.

### 2.3 Sample size

According to Epskamp and Fried’s tutorial thesis (30), a minimum sample size of P(P-1)/2 is required to ensure sufficient statistical power for a partial correlation network consisting of P nodes. In our study, P represented 26 symptoms (nodes), indicating that the sample size should be at least 325. In our study, we had a total of 586 participants, exceeding the required sample size and ensuring the reliability of our results.

### 2.4 Measures

#### 2.4.1 Reported symptoms

The Memorial Symptom Assessment Scale 10-18 (MSAS 10–18) was derived from the adult version of the MSAS (31), which was adapted by Collins et al. in 2000 (8). The scale was used to evaluate the past week’s symptoms in children with cancer aged 10–18 years. Later, some studies found that the scale was applicable to the symptom experience of children aged 1–18 years through proxy reporting (32, 33). The scale exhibits comprehensiveness regarding its dimensions and ratings of each symptom. It is widely utilized to evaluate the symptom burden in children with cancer and has demonstrated validity in national and international cohorts (9, 34, 35). The Chinese version of the scale was translated and cross-culturally adapted by Feng et al. in 2012, and its Cronbach alpha coefficient was 0.897 (36). The MSAS 10–18 comprises 31 items; 23 items evaluate the prevalence, frequency, and severity of symptoms, and 8 items assess their incidence, severity, and distress levels. The symptoms are rated with “ yes ” or “ no ” binary responses. They are further measured by their frequency and severity using a 4-point Likert scale (1 = “ rare ” to 4 = “ almost always ” for frequency, 1 = “ slight ” to 4 = “ very severe ” for severity). Similarly, distress is evaluated using a 5-point Likert scale (0 = “ none at all ” to 4 = “ very much ”), and a composite symptom score is obtained as the mean value of scores obtained across various dimensions of the symptom. The Cronbach’s alpha coefficient for the MSAS 10–18 in this study was 0.814, indicating good internal consistency.

#### 2.4.2 Sociodemographic and clinical data

Demographic, socioeconomic, and clinical data were collected from a self-administered questionnaire. Demographic factors included age (continuous), sex (male, female), being the only child (yes, no), residence (urban, rural) and the primary caregiver (parents, others). Clinic data had clinical classification (standard risk, intermediate risk and high risk), chemotherapy phase (induction, intensified consolidation and maintenance phases), and chemotherapy sessions (number of times).

### 2.5 Data collection

The ethical approval for the study was obtained from the Institutional Ethics Board of Guangzhou Women and Children’s Medical Center, China (IRB 2021233A01). Three research team members collected all data at a tertiary teaching hospital’s inpatient and day ward between January 2021 and April 2023. They were trained to review medical records and collect participants’ demographic data. Participants who met the inclusion criteria completed the questionnaire after providing written informed consent. The children and primary caregiver were assented to before the investigation and subsequently received guidelines for completing the questionnaire. Typically, children aged eight or above can self-report their symptoms, whereas children aged five to eight can only partially report them (37). Therefore, for children aged below eight, the primary caregiver assists them in reporting their symptoms. For respondents who could not comprehend the contents of the questions, we conducted face-to-face interviews in Mandarin or another relevant dialect to gather the data. After completing the questionnaire, each participant received a small incentive for participation. The survey was administered to eligible participants through paper-based questionnaires and an online follow-up platform. Three researchers assessed the questionnaire quality. Out of the 612 distributed questionnaires, 8 were invalid due to answering questions in a fixed pattern (eg, 31 consecutive questions with the same answer) and 18 were invalid due to data missing (over 50%). Thus, 586 questionnaires were valid, resulting in an effective response rate of 95.7%.

### 2.6 Statistical analyses

All statistical analyses were conducted using R version 4.1.2 (R Core Team, 2021; https://www.r-project.org/; Vienna, Austria). Descriptive statistics, including frequencies, percentages, means, and standard deviations (SD), were utilized to describe the demographics and severity of symptoms. To ensure the stability of clusters and symptom networks, only symptoms with a prevalence of over 20% were included to ensure the stability of symptom clusters, resulting in the inclusion of 26 symptoms for identifying symptom clusters and networks (38, 39). Principal component analysis (PCA) was conducted using the R Psych package to identify the dimensions of the symptoms (mean of multiple dimensions symptom score). The number of factors was determined based on a scree plot in the factor analysis. In the PCA, an orthogonal transformation (varimax rotation) was utilized, and factors with Eigen values greater than 1.0 were included. Horn’s parallel analysis was used to confirm the number of factors. Cronbach’s alpha coefficient was used to evaluate the internal consistency and reliability of principal components, and only symptoms with factor loadings greater than 0.45 were included in clusters (24). Symptom clusters consisted of at least two symptoms, with the symptom having the higher factor loading being assigned to the corresponding cluster if it appeared in multiple clusters. We used Cronbach’s alpha coefficient to assess the internal consistency and reliability of the derived factors. Finally, we conducted a team discussion to ensure the clinical relevance of the derived symptom clusters.

The relationships among 26 symptoms and clusters were examined using an association network analysis conducted with the R package, *qgraph*. Spearman correlations were used to estimate the relationships between symptom pairs (symptom score) and symptom clusters (standardized symptom score) in the symptom network. An undirected association network was created using the spring layout algorithm, placing nodes with stronger connections closer to the network’s center. The edges in the network indicated conditional independence between nodes, with thicker edges indicating stronger associations between the connected nodes. To reduce false positives, we used the least absolute shrinkage and selection operator (LASSO) to remove small edges and applied the extended Bayesian information criterion (EBIC) with tuning parameter γ to achieve an optimal network fit (30, 40). Owing to using orthogonal transformation in principal component analysis, which may minimize the impact of individual items, we included all symptom clusters and individual symptoms in the network analysis to detect centrality indices (24). Centrality indices, including strength, betweenness, and closeness, were used to assess the importance of nodes and symptom clusters in the network. Strength represents the frequency of co-occurrence between a symptom and others, with higher values indicating more frequent connections. Betweenness measures the extent to which a symptom connects other nodes in the shortest path, indicating its impact on the overall network. Closeness is determined by the average distance between a symptom and all other nodes, with shorter paths corresponding to higher values of closeness.

Multiple linear regression models were used in this study to analyze factors associated with symptom scores in children with AL. The model included the following demographic and clinical variables: sex (male = 0, female = 1), age (continuous), being the only child (no = 0, yes = 1), place of residence (urban = 0, rural = 1), the primary caregiver (parents = 0, otherwise = 1), monthly per capita household income (below 4000 Chinese Yuan = 0, otherwise = 1), education degree of the primary caregiver (high school and below = 0, otherwise = 1), marriage status of the primary caregiver (married = 0, otherwise = 1), clinical classification (standard risk = 0, intermediate risk and above = 1), chemotherapy phase (induction phase = 0, otherwise = 1), chemotherapy sessions (number of times). A two-tailed P value less than 0.05 indicated statistical significance in all analyses. We used multiple imputations to handle missing data for three variables (clinical classification, chemotherapy sessions, and marital status of primary caregiver). This was done by creating five imputed data sets using multivariate imputation by chained equations (implemented through the R package mice). The imputed data sets were selected to closely resemble the original data distribution (41, 42).

## 3 Results

### 3.1 Participant characteristics

A total of 586 participants were analyzed in this study. **Table 1** shows the characteristics of the participants. Most participants were male (n = 367, 62.6%), were an only child (n = 182, 31.1%), lived in rural (n = 340, 58.0%), were intermediate risk in clinical classification (n = 329, 56.1%), were in induction and remission phase (n = 264, 45.1%). The participants had a mean (SD) age of 8.29 (2.42) years and underwent an average of 4.25 (3.11) chemotherapy sessions. The primary caregiver was a parent (n = 552, 94.2%), had a post-high school education level (n = 152, 25.9%), was 2000-3999 Chinese Yuan (approximately 286-571 United States Dollars (USD)) monthly per capita household (n = 223, 38.1%), was married (n = 564, 96.2%).

**Table 1:** Participant characteristics (N = 586).

Characteristics	n (%), Mean (SD)
Sex	
Male	367 (62.6)
Female	219 (37.4)
Age	8.29 (2.42)^a^
Only child	182 (31.1)
Residence	
Urban	246 (42.0)
Rural	340 (58.0)
Primary caregiver	
Parents	552 (94.2)
Others	34 (5.8)
Education degree of primary caregiver	
Primary school or below	145 (24.7)
High school	139 (23.7)
Post-high school	152 (25.9)
University or above	150 (25.6)
Marriage status of the primary caregiver	
Married	564 (96.2)
Single or bereaved a spouse	22 (3.8)
Monthly per capita household income (Chinese Yuan)^b^	
< 1000 (approximately 143 USD)	28 (4.8)
1000-1999 (approximately 143-286 USD)	149 (25.4)
2000-3999 (approximately 286-571 USD)	223 (38.1)
4000-5000 (approximately 571-714 USD)	157 (26.8)
> 5000 (approximately 714 USD)	29 (4.9)
Clinical classification	
Standard risk	142 (24.2)
Intermediate risk	329 (56.1)
High risk	115 (19.6)
Chemotherapy phase	
Induction and remission	264 (45.1)
Consolidation and intensification	179 (30.5)
Maintenance treatment	143 (24.4)
Chemotherapy sessions	4.25 (3.11)^a^

a The continuous variable is characterized by its mean and standard deviation.

b The exchange rate between the Chinese yuan and the US dollar is based on 1:7.

### 3.2 Prevalence and score of symptoms

**Table 2** shows the prevalence and score of the participant’s symptoms. Regarding the prevalence of symptoms, lack of energy (n = 391, 83.4%) was the most common symptom, followed by pain (n = 374, 79.7%), hair loss (n = 344, 73.3%), feeling sad (n = 340, 72.5%) and worrying (n = 335, 71.4%). Regarding the score of symptoms, pain (mean = 1.86, SD = 1.16) was the most severe symptom, followed by lack of energy (mean = 1.80, SD = 1.05), worrying (mean = 1.55, SD = 1.21), feeling sad (mean = 1.48, SD = 1.08) and hair loss (mean = 1.47, SD = 1.10).

**Table 2:** Prevalence and Score of symptoms (N = 586).

Variates	Prevalence (n, %)	Score (0–4) Mean (SD)
First part		
Lack of concentration	309 (65.9)	1.25 (1.07)
Pain	374 (79.7)	1.86 (1.16)
Lack of energy	391 (83.4)	1.80 (1.05)
Cough	236 (50.3)	1.04 (1.20)
Feeling nervous	317 (67.6)	1.32 (1.08)
Dry mouth	202 (43.1)	0.83 (1.07)
Nausea	275 (58.6)	1.24 (1.18)
Feeling drowsy	211 (45.0)	0.70 (0.88)
Tingling in hands or feet	183 (39.0)	0.70 (1.00)
Insomnia	269 (57.4)	1.18 (1.16)
Problems with urination	34 (7.2)	0.09 (0.38)
Vomiting	241 (51.4)	1.11 (1.21)
Dyspnea	84 (17.9)	0.25 (0.58)
Diarrhea	227 (48.4)	0.85 (0.99)
Feeling sad	340 (72.5)	1.48 (1.08)
Sweating	146 (31.1)	0.61 (1.01)
Worrying	335 (71.4)	1.55 (1.21)
Itching	215 (45.8)	0.84 (1.04)
Lack of appetite	138 (29.4)	1.26 (1.17)
Dizziness	60 (12.8)	0.23 (0.67)
Difficulty swallowing	46 (9.8)	0.14 (0.47)
Feeling irritable	207 (44.1)	0.93 (1.21)
Cephalalgia	78 (16.6)	0.26 (0.63)
Second part		
Mouth sores	216 (46.1)	1.04 (1.22)
Change in the way food tastes	213 (45.4)	0.95 (1.15)
Weight loss	139 (29.6)	0.41 (0.71)
Hair loss	344 (73.3)	1.47 (1.10)
Constipation	135 (28.8)	0.49 (0.84)
Swelling of arms or legs	124 (26.4)	0.48 (0.89)
I don’t look like myself	266 (56.7)	1.02 (1.04)
Changes in skin	159 (33.9)	0.52 (0.83)

### 3.3 Symptom networks and centrality indices

**Figure 1:**
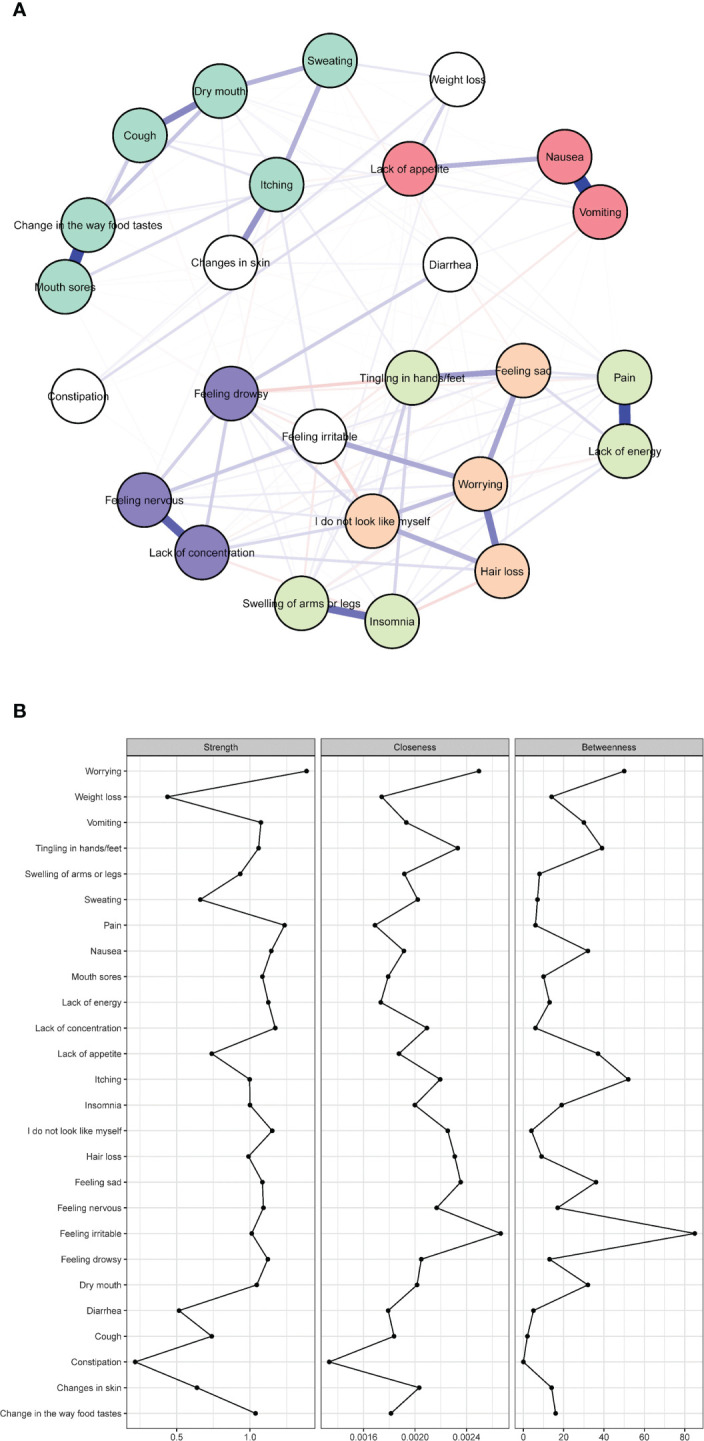
Network of symptoms and centrality indices. (N = 586). **(A)** Symptom network of 26 symptoms; **(B)** strength, betweenness, and closeness of 26 symptoms; The red color represents the gastrointestinal symptom cluster, green represents the oral and skin symptom cluster, orange represents the self-image symptom cluster, indigo represents another physical symptom cluster, purple represents the psychological symptom cluster, and white represents individual symptoms not included in any specific symptom cluster.

**Figure 1A** presents symptom network and centrality indices among 26 symptoms. The three strongest edges were between “ nausea ” and “ vomiting ” (*r* = 0.80), “ mouth sore ” and “ change in the way food tastes ” (*r* = 0.71) and “ lack of energy ” and “ pain ” (*r* = 0.68). **Figure 1B** display three centrality indices: strength, closeness, and betweenness. In the symptom network, worrying (*r*_s_ = 1.45, *r*_c_ = 0.00, *r*_b_ = 52) indicated the largest values for strength; “ feeling irritable ” (*r*_s_ = 1.26, *r*_c_ = 0.00, *r*_b_ = 96) showed the largest value for betweenness and closeness.

### 3.4 Prevalence and composition of symptom clusters

**Table 3** shows the factor loading of each symptom and the resulting symptom clusters. The Kaiser measure of sampling adequacy was high for the PCA (Kaiser-Meyer-Olkin = 0.740). The following five symptom clusters had Eigen values greater than 1.0 and above the intersection of two lines in the parallel analysis: 1) oral and skin; 2) somatic; 3) self-image disorder; 4) gastrointestinal; and 5) psychological. Seven symptoms, including diarrhea, feeling irritable, weight loss, constipation, and change in the skin, had low loading on all factors. The most common symptom cluster was self-image disorder (90.61%), followed by somatic (89.76%), oral and skin (78.67%), psychological (77.30%), and gastrointestinal (73.35%). The Cronbach’s α coefficient values showed acceptable internal consistency among the five derived symptom clusters.

**Table 3:** Summary of cluster symptoms.

Cluster	Cluster Composition	Factor Loading	Prevalence	Cronbach’s Alpha
Oral and skin	Cough	0.740	461 (78.67)	0.85
	Dry mouth	0.754		
	Sweating	0.585		
	Itching	0.670		
	Mouth sores	0.831		
	Change in the way food tastes	0.800		
Somatic	Pain	0.798	526 (89.76)	0.80
	Lack of energy	0.812		
	Tingling in hands or feet	0.734		
	Insomnia	0.628		
	Swelling of arms or legs	0.687		
Self-image disorder	Feeling sad	0.581	531 (90.61)	0.77
	Worrying	0.807		
	Hair loss	0.777		
	I do not look like myself	0.735		
Gastrointestinal	Nausea	0.873	424 (73.35)	0.85
	Vomiting	0.835		
	Lack of appetite	0.735		
Psychological	Lack of concentration	0.833	453 (77.30)	0.76
	Feeling nervous	0.821		
	Feeling drowsy	0.617		

### 3.5 Symptom network of clusters and symptoms

**Figure 2:**
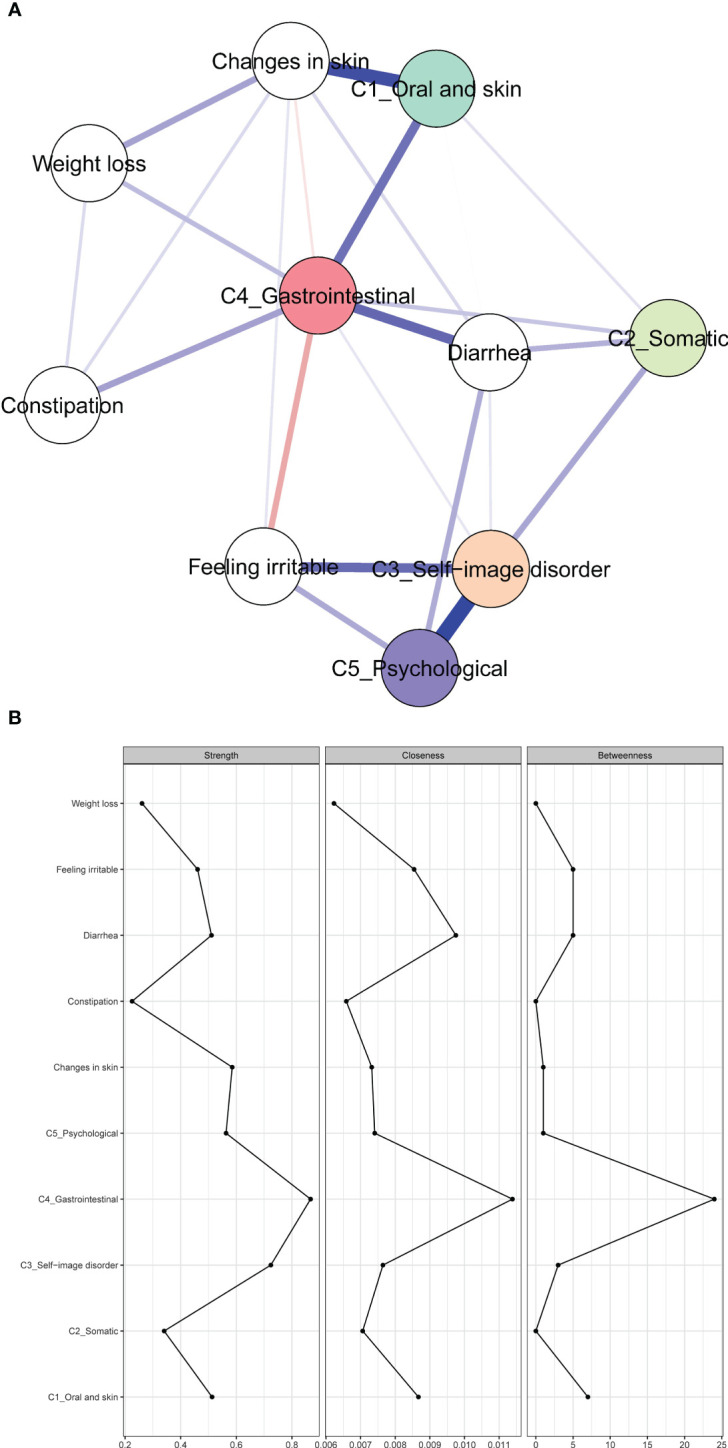
Network of symptoms and symptom clusters, and centrality indices. **(A)** Network of symptoms and symptom clusters. **(B)** Strength, betweenness, and closeness of symptoms and clusters.

**Figure 2** shows the association network and centrality indices among the five symptom clusters and seven symptoms. The three strongest edges were between “ self-image disorder ” and “ psychological ” (*r* = 0.34), “ oral and skin ” and “ changes in skin ” (*r* = 0.28), and “ gastrointestinal ” and “ diarrhea ” (*r* = 0.20). In the entire network, “ gastrointestinal ” (*r*_s_ = 0.87, *r*_c_ = 0.01, *r*_b_ = 24) was the most central symptom cluster across the three centrality indices, followed by “ self-image disorder ” (*r*_s_ = 0.72, *r*_c_ = 0.01, *r*_b_ = 3), and “ oral and skin ” (*r*_s_ = 0.51, *r*_c_ = 0.01, *r*_b_ = 7).

### 3.6 Multiple linear regression models of overall symptoms and symptom clusters

The results of the exploratory multiple linear regression models of overall symptoms and five symptom clusters are shown in **Table 4**. Participants who were younger (*P* < 0.010), received chemotherapy during the induction phase (*P* < 0.001) and had undergone fewer chemotherapy sessions (*P* < 0.001) were more likely to report higher overall symptom scores. Variables including sex, age, education degree of primary caregiver, chemotherapy phase, chemotherapy sessions, and clinical classification were associated with the severity of five symptom clusters.

**Table 4:** Multiple linear regression of five symptom clusters (N = 586).

Variable	Model 1 Overall	Model 2 Oral and skin	Model 3 Somatic	Model 4 Self-image disorder	Model 5 Gastrointestinal	Model 6 Psychological
Intercept	35.548^c^	1.078^c^	1.687^c^	1.537^c^	1.599^c^	1.570
Male (compared to female)	-1.243	0.037	-0.044	-0.150^a^	-0.053	-0.250^c^
Age	-0.665^b^	-0.023	-0.021	-0.035^a^	-0.020	-0.036^a^
Living in urban (compared to living in rural)	0.161	-0.034	0.085	-0.011	0.021	0.043
Having an only child (compare to two or more children)	-1.675	-0.032	-0.129	-0.092	-0.016	-0.145
primary caregiver during chemotherapy: parent (compare to otherwise)	-0.457	0.009	-0.093	-0.011	-0.163	0.121
Education degree of primary caregiver: high school and below (compare to otherwise)	1.951	-0.029	0.071	0.301^c^	0.198	0.100
Marriage status of primary caregiver: married (compare to single)	-1.650	0.368	-0.244	-0.674^c^	0.125	0.035
Monthly per household income: below 4000 Chinese Yuan (compare to otherwise)	-0.042	0.087	-0.090	-0.089	-0.066	-0.032
Standard risk of clinical classification (compare to otherwise)	-2.312	-0.055	-0.233^b^	-0.089	-0.058	-0.025
During induction phase of chemotherapy (compare to otherwise)	5.821^c^	0.369^c^	0.108	0.370^c^	0.261^a^	0.118
Number of having chemotherapy	-0.899^c^	-0.053^c^	-0.042^b^	0.002	-0.097^c^	-0.034^a^

Model 1: *F* = 4.435, *P* < 0.001, ***R** ^2^_adj_* = 0.061; Model 2: *F* = 2.264, *P* =0.001, ***R** ^2^_adj_* =0.023; Model 3: *F* = 3.438, *P* < 0.001, ***R** ^2^_adj_* =0.044; Model 4: *F* = 7.669, *P* < 0.001, ***R** ^2^_adj_* =0.111; Model 5: *F* = 3.378, *P* < 0.001, ***R** ^2^_adj_* =0.043; Model 6: *F* = 2.843, *P* < 0.001, ***R** ^2^_adj_* = 0.034.

a *P* < 0.05.

b *P* < 0.01.

c *P* < 0.001.

## 4 Discussion

This study is the first to identify central symptom clusters and explore the relationships among symptoms in the symptom networks, using the network analysis in children with AL undergoing chemotherapy. Five symptom clusters were identified, including oral and skin cluster, somatic cluster, self-image disorder cluster, gastrointestinal cluster, and psychological cluster. In the whole symptom network, the gastrointestinal cluster was the most central symptom cluster across the three centrality indices (strength, closeness, and betweenness). Age, sex, clinical classification, number of having chemotherapy and education degree and marital status of the primary caregiver are associated with the severity of these five symptom clusters.

Five symptom clusters were identified from the data. Cluster 1, consisting of oral and skin symptoms, demonstrated an overlap of cough, dry mouth, sweating, itching, mouth sores, and change in the way food tastes. In contrast to previous studies that identified an oral-related cluster (11, 17), we observed the inclusion of dry mouth and itching symptoms. Therefore, we labeled this cluster as “ oral and skin ” due to the presence of symptoms related to both the oral and skin regions. This cluster of symptoms, including dry mouth and itching, is likely related to the effects of chemotherapy drugs. Dry mouth is caused by chemotherapy’s impact on the salivary glands, reducing saliva production and increasing susceptibility to oral infections (43–45). Additionally, methotrexate can cause oral mucositis, leading to mouth sores and pain (46). During the induction phase, children with AL experienced higher severity of the oral and skin symptom cluster compared to other phases, possibly due to the higher chemotherapy dose administered to achieve complete remission (11). Therefore, it is important for healthcare providers to assess the oral and skin cluster during the induction phase.

Cluster 2, characterized as somatic symptoms, displayed an overlap of pain, lack of energy, tingling in hands or feet, insomnia, and swelling in the arms or legs. Consistent with previous studies, fatigue (lack of energy), pain, insomnia, and depression were identified as the most significant symptom clusters in cancer survivors (13, 47, 48). Our study has revealed that the peripheral nerve dysfunction of limbs (i.e., tingling in hands or feet and swelling of arms or legs) also consist of this cluster. However, unlike a previous study (11), we did not find an association between the chemotherapy phase and somatic symptoms. This discrepancy may be attributed to variations in sample characteristics, study populations, and statistical methods employed. Consistent with a previous study (29), our findings demonstrated that children of AL with intermediate and high-risk classifications might experience greater severity of somatic symptoms compared to those with standard risk. Not surprisingly, children classified as intermediate and high-risk face multiple risk factors, including age, high initial leukocyte count, immunophenotype, cytogenetic/genomic alterations, extramedullary disease, Down syndrome, and minimal residual disease (MRD > 1%) after induction (49, 50). Therefore, these intermediate and high-risk children with AL undergo intensified consolidation therapy, leading to more severe physical symptoms. Furthermore, in line with previous studies (32, 38), we observed a decrease in the severity of somatic symptoms across successive chemotherapy sessions. These findings are expected, as most children with acute leukemia undergo induction and remission chemotherapy, leading to the alleviation or disappearance of most physical symptoms (51). As treatment continues, the severity of symptoms progressively decreases. Based on our findings, we recommend that clinical care providers prioritize children diagnosed with acute leukemia who have intermediate risk and above, particularly during the early stages of chemotherapy.

Cluster 3 was self-image disorder, which showed an overlap among feeling sad, worrying, hair loss, and “ I do not look like myself.” In our study, the self-image disorder cluster was the most prevalent in children with acute leukemia. Hair loss and “ I do not look like myself ” were consistent with previous studies (11, 16, 17), but our study also included worrying and feeling sad in this cluster, possibly due to the emotional impact of hair loss in children (11). In line with a previous study (11), girls may exhibit more pronounced symptom clusters, likely due to their increased focus on changes in appearance. However, contrary to a previous finding (11), we did not observe a significant association between older age and self-image cluster. This discrepancy may be attributed to differences in the study population. Interestingly, we identified a correlation between low caregiver education levels, single marital status, and symptom clusters in children with AL. This may be explained by caregivers feeling reluctant to express emotions and a sense of identity towards their children based on personal experiences or cultural backgrounds of China (i.e., conserved and modest) that induce feelings of shame or reluctance. Therefore, healthcare providers managing the self-image symptom cluster should not only focus on the children but also prioritize their primary caregivers, especially those with lower educational attainment and single-parent status.

Cluster 4 represents gastrointestinal symptoms. This study found that the gastrointestinal symptom cluster consisted of nausea, vomiting, and appetite loss, which was a stable and consistent combination that did not vary with cancer type, symptom assessment tools, or statistical methods used (11, 13, 16). Consistent with a prior study (11), the severity of gastrointestinal symptoms was higher in the induction phases, which was associated with the chemotherapy phase. This result may be due to the sequence of chemotherapy regimens, where the early stages of leukemia treatment have a higher drug dosage, resulting in higher severity of this symptom cluster in the early stages (11). Inconsistent with previous studies (11, 29), we found that the lower education level of the primary caregiver was associated with a more severe symptom cluster. The potential reason is that primary caregivers with higher education possess superior knowledge of healthy diets and the prescribed dietary guidelines recommended by doctors during chemotherapy. The primary cause of gastrointestinal reactions is chemotherapy drugs stimulating chromaffin cells to release 5-hydroxytryptamine, thereby triggering the emetic center (52). Furthermore, methotrexate can decrease plasma levels of cysteine, increasing cell sensitivity to oxidative stress and resulting in gastrointestinal mucosal damage, inflammation, and adverse effects such as nausea and vomiting (53).

Additionally, we found that gastrointestinal symptom cluster have the highest values across three centrality indices, showing that this cluster was the most central symptom cluster in the symptom network. A central symptom may spread the intervention effects to the peripheral nodes of the central symptom, eventually leading to the remission or disappearance of the other symptoms (23). In our study, gastrointestinal symptom cluster is associated with a somatic cluster, oral and skin cluster and self-image disorder cluster. Based on network theory, targeting central symptoms in intervention treatment can accelerate the deactivation of the symptom network and improve the precision and efficiency of the intervention (23). Therefore, targeting gastrointestinal symptom clusters is imperative to remiss the symptom burden (23). According to a previous study, the prevalence of malnutrition, as defined by the WHO to include both undernutrition and overnutrition, can reach up to 75% among children and adolescents with cancer (54). It is linked to lower survival rates, increased toxicity risks, reduced treatment tolerance, and higher infection susceptibility (55, 56). Therefore, based on the results of this study, early prevention and detection of malnutrition in children with cancer are crucial. Clinical healthcare professionals can establish a multidisciplinary nutrition support team consisting of a nutritionist, specialized nurse, and physician to provide individualized nutrition support for children with severe gastrointestinal symptoms and malnutrition. Nutritional status assessment and risk stratification should be conducted before chemotherapy, and treatment-related adverse reactions should be promptly evaluated and managed. Drug therapies such as dopamine receptor antagonists, trimethobenzamide, 5-HT3 receptor antagonists and 5-HT4 receptor agonists have been shown to be effective in addressing nausea and vomiting (57). Additionally, complementary and alternative medical therapies, such as ginger (58), acupuncture (59), behavioral therapy (60) and virtual reality (61–63), have also demonstrated effectiveness in managing nausea and vomiting symptoms.

Cluster 5 was the psychological symptom, which showed an overlap among lack of concentration, feeling nervous, and feeling drowsy. Similar to previous studies, the presence of lack of concentration and feelings of nervousness constitutes an essential component of the psychological symptom cluster (11, 16, 17). However, we observed that younger ages and girls were associated with more severe psychological clusters, particularly in terms of emotional symptoms. The severity of girls’ emotional cluster was rated higher than boys, which may be due to girls being more willing to admit negative emotions than boys or girls being more likely to express mental states such as nervousness (11). In addition, we found that low age is associated with a more severe psychology cluster. A prior study by Baggott et al., which involved 107 pairs of parents and children with cancer, aimed to examine the agreement in symptom reporting (64). It was found that parents tended to overestimate the severity of psychosocial concerns, such as lack of concentration and nervousness, based on their levels of distress or expectations (64). In our study, we assessed symptom severity in children below 8 years old using parent proxy reports. It’s important to consider that these reports may potentially overestimate the severity of symptoms, as they rely on parental reporting rather than direct assessment.

## 5 Strength and limitations

This study stands as the pioneering research to identify central symptom clusters in children with acute leukemia undergoing chemotherapy. Additionally, it offers valuable empirical evidence to aid healthcare providers and researchers in developing precision and personalized care approaches. There are certain limitations in our study. First, the cross-sectional design and convenience sampling methodology restrict the findings’ generalizability, and causal relationships between symptoms and clusters could not be determined. A longitudinal study is imperative to explore the dynamic network and identify symptoms and clusters that adversely affect other symptoms and clusters. Second, caregivers reported some of the children’s symptoms in our study. Caregiver proxies were used for children younger than eight years of age. They may be less reliable for less easily observed symptoms (e.g., lack of concentration, nervousness). Furthermore, our study did not analyze different age groups, limiting the generalizability of our findings to children of various ages. Future research should investigate symptom clusters across different age ranges to enable personalized care.

## 6 Implication for research and clinical practice

This study’s main findings of identifying five distinct symptom clusters (oral and skin, somatic, self-image disorder, gastrointestinal, and psychological) have important implications for research and clinical practice in pediatric oncology. The central role of the gastrointestinal cluster highlights the need for targeted interventions in managing symptom clusters. Additionally, demographic and clinical factors such as age, sex, clinical classification, chemotherapy sessions, caregiver education and marital status are associated with the severity of these clusters.

These findings emphasize the importance of personalized care in pediatric oncology, considering individual characteristics when developing interventions. They contribute to understanding symptom clusters and can guide comprehensive symptom assessment and management strategies. Future research should explore underlying mechanisms and longitudinal changes in these clusters to improve treatment outcomes and QoL.

In clinical practice, healthcare providers can utilize these findings to optimize care for children with acute AL undergoing chemotherapy. By addressing specific symptom clusters and considering individual factors, interventions can be tailored to improve symptom management and enhance the overall care experience for these young patients.

## 7 Conclusion

This study contributes new insights into symptom clusters in children with acute leukemia. identifying five symptom clusters: oral and skin cluster, somatic cluster, self-image disorder, gastrointestinal cluster, and psychological cluster. Gastrointestinal cluster was the most central symptom cluster. Our study underscores the complex and varied nature of symptom distress in children with acute leukemia undergoing chemotherapy. It emphasizes the importance of evaluating symptom clusters in this context. Specifically, addressing gastrointestinal symptoms is crucial for effective symptom management and overall care.

## Data availability statement

The raw data supporting the conclusions of this article will be made available by the authors, without undue reservation.

## Ethics statement

The ethical approval for the study was obtained from the Institutional Ethics Board of Guangzhou Women and Children’s Medical Center (IRB 2021233A01), and the study was conducted in compliance with the Declaration of Helsinki. Before data collection, written informed consent was obtained from all participants and the primary caregiver.

## Author contributions

JF and C-QL: literature review and manuscript drafting. H-YH, L-LX, and M-XW: data acquisition. C-LW, Y-SQ, and YL: study design and manuscript revision. All authors contributed to the article and approved the submitted version.
